# Yield, Nutritional, and Thermal Responses of Lettuce (*Lactuca sativa*) and Eggplant (*Solanum melongena*) Under Greenhouse Covers with Different UV-B Transmittance

**DOI:** 10.3390/plants15060863

**Published:** 2026-03-11

**Authors:** Mauro Mori, Eugenio Cozzolino, Ida Di Mola, Lucia Ottaiano, Antimo Di Meo, Pasquale Mormile, Massimo Rippa

**Affiliations:** 1 Department of Agricultural Sciences, University of Naples Federico II, 80055 Portici, Naples, Italy; mori@unina.it (M.M.); ida.dimola@unina.it (I.D.M.); lucia.ottaiano@unina.it (L.O.); 2 Research Center for Cereal and Industrial Crops, Council for Agricultural Research and Economics (CREA), 81100 Caserta, Caserta, Italy; eugenio.cozzolino@crea.gov.it; 3 Institute of Applied Sciences and Intelligent Systems “E. Caianiello” of National Research Council of Italy (CNR ISASI), 80072 Pozzuoli, Naples, Italy; antimo.dimeo1998@gmail.com (A.D.M.); pasquale.mormile@isasi.cnr.it (P.M.)

**Keywords:** UV-B, greenhouse covering, lettuce, eggplant, infrared thermography, metabolites, nutritional response, yield

## Abstract

Ultraviolet-B (UV-B) radiation plays a pivotal role in plant growth, metabolism, and the accumulation of bioactive compounds, but its effects under greenhouse conditions are highly species- and dose-dependent. This study investigated the responses of eggplant (*Solanum melongena* L., cv. Lunga Napoletana) and lettuce (*Lactuca sativa* L., cv. Rosplus) cultivated under greenhouse films transmitting 3–39% of ambient UV-B. Leaf temperature was monitored throughout the growth cycle using infrared thermography, while physiological parameters (chlorophyll, flavonoids, anthocyanins, and nitrogen index) and post-harvest nutritional traits (antioxidant activity, vitamin C, carotenoids, and total chlorophyll) were assessed. Comparative analysis revealed species-specific responses. Eggplant exhibited peak nutraceutical quality at higher UV-B levels (35–39%) with minimal changes in yield, whereas lettuce achieved maximal yield and secondary metabolite accumulation under intermediate UV-B (30–35%). At the highest UV-B transmittance (39%), both species exhibited stable or slightly reduced thermal and physiological parameters, indicating dose-dependent regulatory mechanisms that maintain photoprotection and metabolic activity under elevated UV-B exposure. Results suggest an apparent optimal range of UV-B transmittance in greenhouse systems under the tested experimental conditions, contributing to improved crop productivity and nutritional quality.

## 1. Introduction

In recent years, ultraviolet-B (UV-B) radiation, conventionally defined as the 280–315 nm spectral region according to the CIE classification [1,2], has gained increasing attention in plant science due to its capacity to modulate plant growth, development, and secondary metabolism [3,4,5,6,7,8,9,10,11,12]. Although UV-B represents only a small fraction of the total solar radiation reaching the Earth’s surface, it can exert significant physiological effects on plants even at natural ambient levels. Under moderate and naturally occurring conditions, UV-B exposure has been shown to stimulate the synthesis of protective secondary metabolites without inducing the detrimental effects commonly associated with high or artificial radiation doses [13,14,15,16]. These secondary metabolites—including flavonoids, carotenoids and anthocyanins—play key roles in plant protection against oxidative stress and environmental variability. At the same time, they contribute substantially to the nutritional and functional quality of edible crops, enhancing their value for human health. The induction of these compounds in response to ambient UV-B radiation is therefore highly relevant from both agricultural and nutritional perspectives. Flavonoids act as powerful antioxidants by scavenging reactive oxygen species and maintaining cellular redox balance, while carotenoids support light capture and photoprotection. Anthocyanins, in addition to their antioxidant capacity, play a key role in UV screening and photoprotection by absorbing excess radiation and mitigating photooxidative damage, while also contributing to the nutritional and functional value of plant-derived foods.

A growing body of literature supports the beneficial role of natural UV-B radiation in improving crop quality. Several studies have demonstrated that exposure to ambient UV-B can stimulate the accumulation of bioactive secondary metabolites in horticultural crops, thereby enhancing antioxidant capacity and nutritional value [17,18,19,20,21]. In this context, UV-B has been increasingly recognized as an effective natural elicitor of secondary metabolism, capable of modulating key biosynthetic pathways even at moderate, non-damaging doses. Indeed, UV-B radiation has been shown to promote phenolic and flavonoid biosynthesis in a wide range of plant species grown under controlled and semi-controlled environments, reinforcing its potential role as a sustainable tool to enhance crop functional quality [22]. Experimental evidence further indicates that the timing and mode of UV-B exposure are critical determinants of plant response. For instance, short-term UV-B exposure applied just before harvest significantly increased the accumulation of flavonoid compounds in both green- and red-leaf lettuce cultivars, resulting in elevated antioxidant activity under controlled environmental conditions [17,23]. These results suggest that even brief exposure to natural UV-B can trigger measurable biochemical responses without negatively affecting crop performance.

Within this context, greenhouse cultivation offers a unique opportunity to regulate crop exposure to natural UV-B radiation. Conventional plastic greenhouse films are typically formulated to block most ultraviolet radiation in order to prevent polymer degradation, a feature that inadvertently limits UV-B-mediated physiological processes in plants. Recent advances in polymer technology, however, have enabled the development of plastic films with controlled and selective UV-B transmittance. These materials create spectral “windows” that allow a defined fraction of ambient UV-B radiation to reach the crop canopy. By selecting appropriate greenhouse coverings, it is therefore possible to modulate UV-B exposure in a controlled manner, potentially enhancing the accumulation of health-promoting secondary metabolites without adversely affecting growth, yield, or developmental timing. Although these films also transmit a portion of UV-A radiation, the effects on secondary metabolite accumulation are largely attributable to UV-B exposure, with potential UV-A effects considered secondary. Greenhouse-based studies provide additional confirmation of the importance of UV-B transmission through covering materials. Early investigations demonstrated that lettuce seedlings grown under plastic films with differing UV-B transmittance exhibited increased flavonoid accumulation when exposed to ambient UV-B, indicating that early-stage acclimation to this spectral component may prime plants for enhanced secondary metabolite production later in development [24]. Subsequent work showed that UV-transparent greenhouse covers promoted higher phenolic content and enhanced leaf coloration in red lettuce cultivars compared with UV-blocking films, highlighting the role of spectral quality in regulating pigment and antioxidant biosynthesis under protected cultivation [25]. Comparable responses have also been observed in other leafy vegetables. Rocket salad (*Eruca vesicaria*) cultivated under greenhouse films transmitting approximately 27% of ambient UV-B exhibited significantly increased concentrations of luteolin and quercetin—two flavonoids with well-established antioxidant properties—relative to plants grown under UV-B-blocking covers [26]. More recent studies have further shown that greenhouse materials with different UV-B transmittance significantly affect both growth-related traits and flavonoid content in lettuce seedlings, underscoring the central role of spectral light quality in regulating plant physiological and metabolic responses [27]. Collectively, these findings underline the specific and distinct role of UV-B radiation, rather than ultraviolet radiation in general, in regulating key metabolic pathways linked to plant quality, defense, and stress resilience. They also emphasize that modulation of UV-B transmittance through greenhouse covering materials represents a practical and agronomically viable strategy to enhance the nutritional and functional attributes of crops under protected cultivation. From an applied perspective, the selective transmission of UV-B through greenhouse coverings represents a practical strategy to enhance both the nutritional and functional properties of crops while maintaining productive performance [16,26]. Nevertheless, plant responses to natural UV-B exposure are strongly species- and cultivar-dependent, influenced by factors such as leaf morphology, pigment composition, and intrinsic photoprotective capacity [14,28]. Moreover, growth stage and environmental conditions can modulate the magnitude of UV-B-induced responses, making empirical evaluation essential for identifying optimal UV-B transmittance levels tailored to specific crops and cultivation contexts [23,29].

Despite the growing interest in exploiting natural UV-B radiation under protected cultivation, comparative studies systematically evaluating species-specific physiological, thermal, and nutraceutical responses under greenhouses with graded UV-B transmittances remain scarce. In particular, there is limited information on how continuous exposure to varying fractions of ambient UV-B affects leaf temperature regulation, flavonoid accumulation, and overall crop functional quality throughout the full growth cycle in horticultural crops. This knowledge gap limits the ability to select greenhouse coverings that simultaneously optimize yield, stress resilience, and nutraceutical quality.

The present study addresses this gap by systematically evaluating the effects of five greenhouse plastic films with different natural UV-B transmittances on eggplant (*Solanum melongena* L., cv. Lunga Napoletana) and lettuce (*Lactuca sativa* L., cv. Rosplus). The experiments were conducted on crops grown in the Acerra area (Campania, Italy), characterized by pedoclimatic conditions typical of the Campanian Plain, within a Mediterranean agro-climatic context. Crop yield, as well as physiological and nutritional traits, were evaluated in both species cultivated under greenhouse coverings transmitting different fractions of ambient UV-B radiation (24–39%) and compared with a reference greenhouse with minimal UV-B transmission.

In addition, leaf temperature was monitored throughout the cultivation cycle at multiple growth stages using non-invasive infrared thermography (IT). Thermal imaging has been widely applied to investigate plant physical and physiological responses under varying environmental and radiative conditions, as well as to detect and characterize biotic and abiotic stresses, because leaf temperature integrates radiation absorption, transpiration, and metabolic regulation [30,31,32,33,34,35,36,37,38,39,40,41,42,43,44,45,46,47,48]. As such, IT provides a valuable, real-time indicator of plant physiological status and its interaction with the greenhouse microclimate over the entire growth period.

The integrated experimental approach adopted in this study aims to: (i) investigate the yield, nutritional, and physiological responses of eggplant and lettuce to prolonged exposure to different levels of naturally transmitted UV-B radiation during their full growth cycle; and (ii) identify specific ranges of UV-B transmittance that optimize crop performance while enhancing nutraceutical quality. By combining agronomic, biochemical, and thermal analyses, this work seeks to provide indications for the selection of greenhouse covering materials capable of exploiting natural UV-B radiation to improve plant productivity and functional food quality under protected cultivation.

## 2. Results and Discussion

Leaf temperature was monitored during four growth stages using IT, while physiological parameters were measured in vivo at two growth stages using a multiparameter device. Nutritional traits were assessed post-harvest. The optical characterization of the plastic films and the results obtained under the five greenhouse covers are presented and discussed in the following sections.

### 2.1. Film Characterization and Experimental Setup

**Figure 1:**
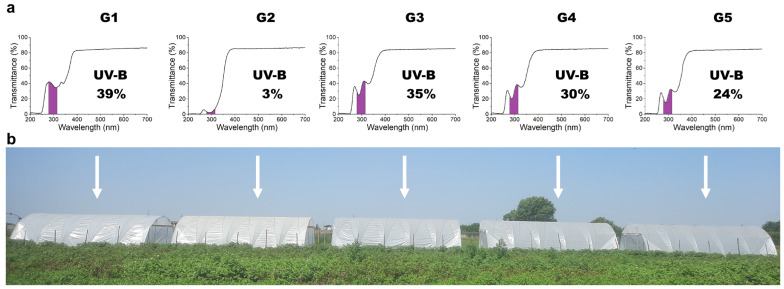
Optical characterization and experimental setup of the greenhouses. (**a**) Spectral transmittance of the five plastic films over 200–700 nm, highlighting the UV-B (280–315 nm) fraction. (**b**) Experimental setup showing the arrangement of the five greenhouses covered with films of different UV-B transmittances (3%, 24%, 30%, 35%, and 39%).

The five greenhouse films differed in UV-B transmittance, ranging from 3% (control) to 39%, while UV-A and photosynthetically active radiation (PAR) transmittances showed smaller variations (54–59% and 84–86%, respectively; values are reported in Supplementary Table S1). Figure 1 shows (a) the measured transmittance spectra of the five films with the estimated UV-B fraction and (b) an image of the experimental field, in which the films were randomly arranged across the greenhouses.

### 2.2. Thermal Responses

Mean leaf temperatures measured under greenhouse covers with different average UV-B transmittances (24%, 30%, 35%, and 39%) were evaluated by IT at four key growth stages (10, 24, 38, and 52 Days After Transplanting, DAT) for both eggplant and lettuce. The corresponding absolute temperature values are reported in the Supplementary Data (Tables S2 and S3).

**Figure 2:**
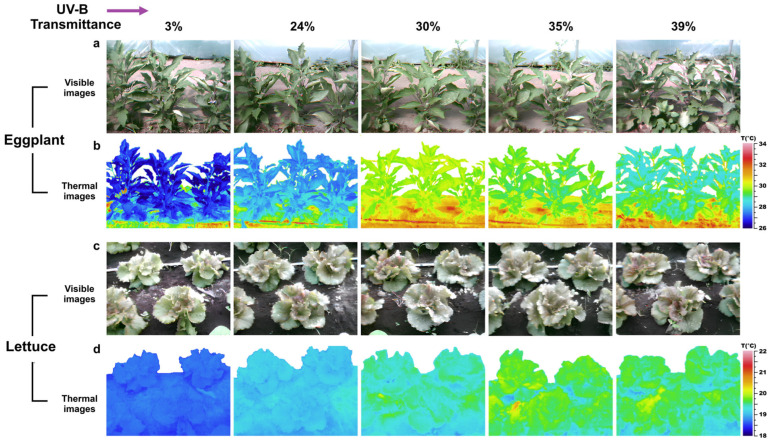
Visual and thermal imaging of eggplant and lettuce under different UV-B transmittances. Representative visible (**a**,**c**) and infrared thermographic (**b**,**d**) images of eggplant (**a**,**b**) and lettuce (**c**,**d**) plants grown under greenhouses with 3%, 24%, 30%, 35%, and 39% UV-B transmittance. Images were captured at 24 Days After Transplanting (DAT).

Representative visible and thermal images of the two crops cultivated under the five greenhouse covers at DAT 24 are reported in Figure 2, providing a visual overview of canopy appearance and leaf temperature variability across the different UV-B treatments.

**Figure 3:**
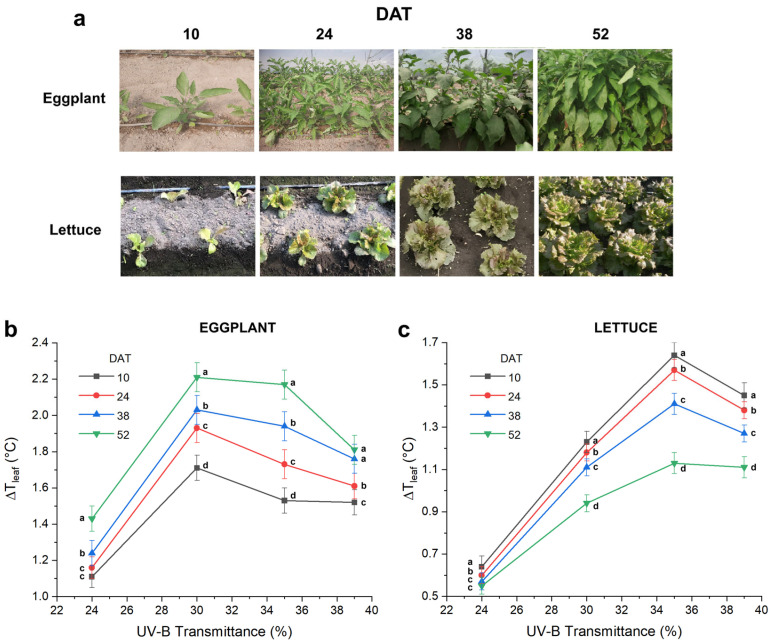
Temporal dynamics of leaf temperature differences (Δ*T_leaf_*) in eggplant and lettuce under varying UV-B transmittances. (**a**) Representative visible images of eggplant (first row) and lettuce (second row) at the four growth stages, illustrating canopy appearance. (**b**) Δ*T_leaf_* in eggplant (*Solanum melongena*, cv. Lunga Napoletana) relative to the reference greenhouse (3% UV-B) at four growth stages (10, 24, 38, 52 DAT). (**c**) Δ*T_leaf_* in lettuce (*Lactuca sativa*, cv. Rosplus) under the same conditions. Different letters indicate statistically significant differences among the four DAT within each UV-B treatment (*p* < 0.05).

Figure 3a shows representative visible images of eggplant (first row) and lettuce (second row) acquired at the four DAT. The corresponding average leaf temperature differences (Δ*T_leaf_*), calculated relative to the reference greenhouse (3% UV-B), are reported for eggplant (Figure 3b) and lettuce (Figure 3c) at the four growth stages. These data allow evaluation of the temporal evolution of plant thermal behavior under increasing UV-B exposure. The results for each crop are discussed in detail below.

On the same DAT corresponding to thermographic monitoring, radiometric variables (UV-B and PAR) were measured inside and outside the greenhouses, together with microclimatic parameters including air temperature (T) and relative humidity (RH). Vapor pressure deficit (VPD) was then calculated from T and RH to provide an additional descriptor of the plant water–atmosphere conditions. These measurements were performed to support the interpretation of the observed thermal patterns and to provide a comprehensive characterization of the radiation and environmental conditions associated with each treatment. Mean values are reported in the Supplementary Material (Tables S4–S11). The results for each crop are discussed in detail below.

#### 2.2.1. Eggplant

Eggplant exhibited a clear and consistent increase in Δ*T_leaf_* with increasing UV-B transmittance across all monitoring dates (Figure 3b). Under 24% UV-B transmittance, Δ*T_leaf_* values increased steadily from 1.11 °C at 10 DAT to 1.43 °C at 52 DAT, indicating a progressive thermal response. The strongest temperature differences were observed under the 30% UV-B-transmitting film, where Δ*T_leaf_* exceeded 2.2 °C at 52 DAT and showed the highest average value across the growth cycle. Interestingly, plants grown under 35% and 39% UV-B transmittance displayed slightly lower than expected Δ*T_leaf_* values, with peaks of 2.17 °C for 35% and 1.81 °C for 39% at 52 DAT, suggesting the activation of adaptive regulatory processes at higher UV-B doses.

It should be noted that, for all four DAT, the average leaf temperatures were slightly higher than the ambient air temperatures within the greenhouses (Table S6). This behavior can be explained by the measured VPD values (Table S8), which were always above 1 kPa during the monitoring periods. Under these conditions, leaf transpiration is somewhat limited, leading to leaf temperatures that are slightly higher than the surrounding air.

#### 2.2.2. Lettuce

Lettuce exhibited overall lower Δ*T_leaf_* values than eggplant across all UV-B treatments and growth stages (Figure 3c), likely due to both seasonal conditions (late autumn–winter cultivation) and species-specific physiological traits. As observed for eggplant, Δ*T_leaf_* increased with UV-B transmittance, but the temporal trend differed markedly. Plants cultivated under the lowest UV-B-transmitting film (24%) exhibited modest temperature differences relative to the control, with Δ*T_leaf_* values ranging from 0.55 °C at 52 DAT to 0.64 °C at 10 DAT, showing a slight decreasing trend over time. In contrast, higher UV-B transmittances resulted in progressively larger Δ*T_leaf_* values, particularly at early and mid-growth stages. The highest average Δ*T_leaf_* was recorded under the 35% UV-B-transmitting film, with values exceeding 1.64 °C at 10 DAT and gradually decreasing to 1.13 °C at 52 DAT. Interestingly, lettuce grown under the highest UV-B transmittance (39%) showed slightly lower Δ*T_leaf_* values than under 35%, ranging from 1.45 °C at 10 DAT to 1.11 °C at 52 DAT, suggesting a non-linear thermal response. This partial saturation indicates that beyond a certain UV-B threshold, lettuce activates compensatory physiological mechanisms that mitigate excessive leaf warming.

It should be noted that, for all four DAT, the average leaf temperatures of lettuce were comparable to, or even lower than, the ambient air temperatures within the greenhouses (Table S9). This behavior can be explained by the measured VPD values (Table S11), which were consistently below 1 kPa during the monitoring periods. Under these low-VPD conditions, leaf transpiration is facilitated, allowing evaporative cooling that can lower the leaf temperature below that of the surrounding air.

### 2.3. Physiological Responses

**Figure 4:**
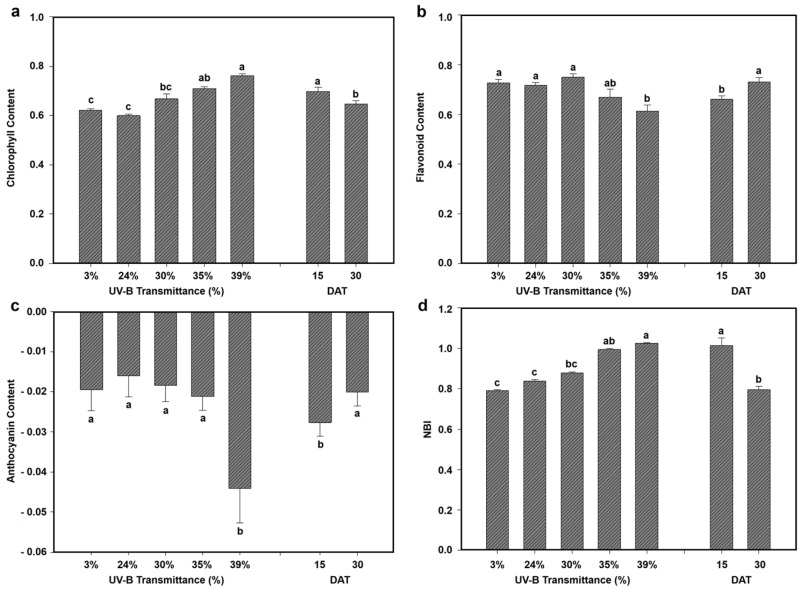
Physiological responses of eggplant to different greenhouse UV-B transmittances. Mean values at 15 and 30 DAT and overall means across all greenhouses are shown for (**a**) chlorophyll content, (**b**) flavonoid accumulation, (**c**) anthocyanin content, and (**d**) nitrogen balance index (NBI). Different letters indicate statistically significant differences among UV-B treatments (*p* < 0.05). Bars represent mean ± SD.

**Figure 5:**
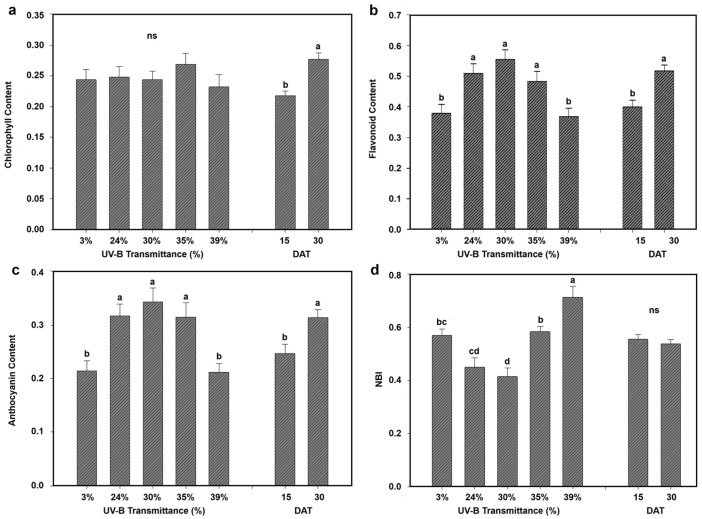
Physiological responses of lettuce to different greenhouse UV-B transmittances. Mean values at 15 and 30 DAT and overall means across all greenhouses are shown for (**a**) chlorophyll content, (**b**) flavonoid accumulation, (**c**) anthocyanin content, and (**d**) nitrogen balance index (NBI). Different letters indicate statistically significant differences among UV-B treatments (*p* < 0.05). Bars represent mean ± SD.

Physiological responses of eggplant and lettuce under the five greenhouse UV-B transmittance levels were evaluated in terms of chlorophyll, flavonoid, anthocyanin content, and nitrogen balance index (NBI). Figure 4 and Figure 5 show the mean values for each treatment across the growth stages, facilitating direct comparison between species and UV-B conditions.

#### 2.3.1. Eggplant

Chlorophyll content in eggplant increased progressively with UV-B transmittance (Figure 4a). Values ranged from 0.62 at 3% UV-B to 0.76 at 39% UV-B, with statistically significant differences among treatments. Mean chlorophyll content was higher at DAT 15 (0.70) than at DAT 30 (0.65), indicating increased photosynthetic pigment accumulation at the earlier growth stage.

Flavonoid accumulation (Figure 4b) showed a UV-B-dependent trend, with maximum values under low-to-intermediate UV-B (30%) and a decrease at higher transmittances (35–39%), reaching the lowest value at 39% UV-B. Mean flavonoid levels were significantly higher at DAT 30 (0.73) than at DAT 15 (0.66), indicating both a UV-B effect and a developmental contribution.

Anthocyanin content (Figure 4c) showed negative values across treatments, indicating undetectable or negligible amounts in eggplant leaves. These values reflect low pigment concentrations near or below the detection limit of the multiparameter device used. Similar observations have been reported for leaves with very low anthocyanin levels, where index values can be small or inconsistent due to the detection limits of non-destructive optical measurements [49,50]. In vegetative tissues with negligible anthocyanin content, these indices do not always strongly correlate with biochemical pigment assays, highlighting the limitations of optical methods under low-pigment conditions.

NBI (Figure 4d) increased markedly with UV-B transmittance, from 0.79 at 3% UV-B to 1.03 at 39% UV-B. Mean NBI was higher at DAT 15 (1.02) than at DAT 30 (0.80), highlighting an early physiological adjustment at the earlier growth stage. This response may reflect changes in nitrogen allocation, protein synthesis, or leaf structural adjustments under UV-B stress.

Overall, eggplant showed a strong and consistent response of chlorophyll and NBI to UV-B transmittance. Flavonoids were moderately modulated, with stabilization or a decline at higher UV-B levels, while the effects on anthocyanin content were minimal or negligible.

#### 2.3.2. Lettuce

In lettuce, chlorophyll content showed limited sensitivity to UV-B transmittance (Figure 5a). Values were relatively stable across all treatments, and no significant differences were observed among the five UV-B levels. Mean chlorophyll content increased with plant age, being higher at DAT 30 (0.28) than at DAT 15 (0.22), indicating a developmental effect rather than a direct UV-B influence.

Flavonoid accumulation (Figure 5b) showed a clear UV-B-dependent response. Intermediate UV-B levels (24–35%) promoted higher flavonoid values compared to both the control (3%) and the highest UV-B (39%). The highest mean flavonoid content was observed at DAT 30 (0.52) versus DAT 15 (0.40), suggesting a combination of UV-B induction and developmental accumulation.

Anthocyanin content (Figure 5c) followed a similar trend, with the highest values at intermediate UV-B levels (24–35%) and a decrease at 39% UV-B. Mean anthocyanin content was significantly higher at DAT 30 (0.31) compared to DAT 15 (0.25), suggesting cumulative induction over time.

NBI (Figure 5d) displayed a non-linear response to UV-B transmittance. Maximum NBI was observed at 39% UV-B, while intermediate levels (24–30%) had lower values. Differences between DAT 15 and DAT 30 were not significant, indicating that nitrogen status in lettuce was more influenced by UV-B transmittance than by plant age.

Overall, lettuce exhibited a more variable physiological response than eggplant. Flavonoids and anthocyanins were strongly induced at intermediate UV-B transmittances, whereas chlorophyll remained stable and nitrogen index increased mainly at the highest UV-B level, indicative of a distinct acclimation strategy compared to eggplant.

### 2.4. Yield and Nutritional Responses

Post-harvest analyses were conducted to assess the effects of greenhouse UV-B transmittance on yield and key nutritional traits in eggplant and lettuce, hydrophilic antioxidant activity (HAA), lipophilic antioxidant activity (LAA), vitamin C, carotenoids, and chlorophyll a + b (Table 1 and Table 2).

#### 2.4.1. Eggplant

In eggplant, yield was not significantly affected by UV-B transmittance, with comparable values observed across all treatments (73.7–85.8 t ha^−1^). In contrast, several nutritional parameters showed a clear and significant response to UV-B exposure. Both antioxidant activities were strongly enhanced under UV-B-transmitting films: LAA increased progressively, reaching a maximum of 17.4 mmol Trolox eq. 100 g^−1^ dw at 35% UV-B (+132% vs. control), while HAA peaked at 5.91 mmol ascorbic acid eq. 100 g^−1^ dw at 39% UV-B (+78% vs. control). Vitamin C content also increased significantly, from 44.5 mg 100 g^−1^ fw in the control to 60.8–64.6 mg 100 g^−1^ fw (+37–45% vs. control) under the highest UV-B transmittances (35–39%). Carotenoids exhibited significant but less consistent variations among treatments, whereas chlorophyll a + b content remained unaffected by UV-B transmittance. For clarity, the complete tables reporting relative changes (% effect sizes) compared to the 3% UV-B reference are provided in Tables S12.

**Figure 6:**
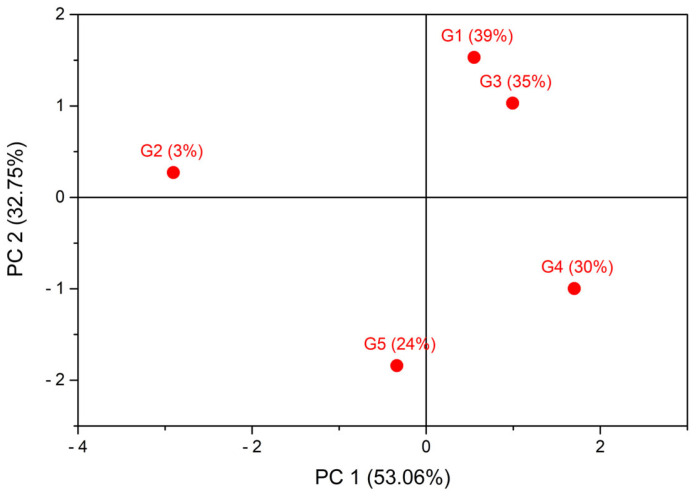
Score plot of the exploratory principal component analysis (PCA) of post-harvest traits in eggplant under five greenhouse UV-B transmittances. Each point represents a greenhouse treatment (G), labeled with its UV-B transmittance (%). PC1 and PC2 explain 53.06% and 32.75% of the total variance, respectively. The plot highlights coordinated, non-linear responses of productivity and nutraceutical traits across the UV-B gradient. Correlation matrices and variable loadings are reported in Supplementary Tables S14 and S15.

To provide an integrated overview of post-harvest responses across the UV-B gradient, a preliminary, exploratory principal component analysis (PCA) was performed using on post-harvest eggplant traits. The PCA score plot (Figure 6) revealed a structured distribution of the five UV-B treatments, supporting the existence of coordinated, though non-linear, responses of productivity and nutraceutical traits.

Correlation matrices and variable loadings are reported in Tables S14 and S15, providing additional detail on trait interrelationships. The first two components explained 53.06% and 32.75% of the total variance, respectively. PC1 primarily separated greenhouses based on carotenoids and yield versus HAA and LAA, indicating a trade-off between pigment accumulation and phenolic antioxidant activity. PC2 was mainly associated with Vitamin C and Chlorophyll a + b, suggesting a partially independent dimension of nutritional quality.

Taken together, these exploratory analyses indicate that moderate to high UV-B transmission substantially enhances the nutritional quality of eggplant fruits— particularly antioxidant capacity (HAA and LAA) and vitamin C content—without causing major changes in yield.

#### 2.4.2. Lettuce

In lettuce, UV-B transmittance significantly influenced both yield and nutritional traits. Yield was maximized under intermediate UV-B transmittance (24–35%), compared to the control (40.8 t ha^−1^, +35%). At the highest UV-B level (39%), yield decreased to 34.2 t ha^−1^ but remained higher than under UV-B-blocking conditions (+13% vs. control), suggesting that excessive UV-B may slightly limit biomass accumulation compared with lower UV-B transmittances. Antioxidant activities were strongly stimulated by UV-B. HAA and LAA reached their maximum values under the 35% UV-B-transmitting film, attaining 7.23 mmol ascorbic acid eq. 100 g^−1^ dw (+44% vs. control) and 7.00 mmol Trolox eq. 100 g^−1^ dw (+79% vs. control), respectively. Vitamin C content increased progressively with UV-B transmittance, reaching a maximum of 40.7 mg 100 g^−1^ fw (+204% vs. control) at 39% UV-B,. Carotenoids and chlorophyll a + b displayed significant but more variable responses, with no clear monotonic trend. For clarity, the complete tables reporting relative changes (% effect sizes) compared to the 3% UV-B reference are provided Tables S13.

**Figure 7:**
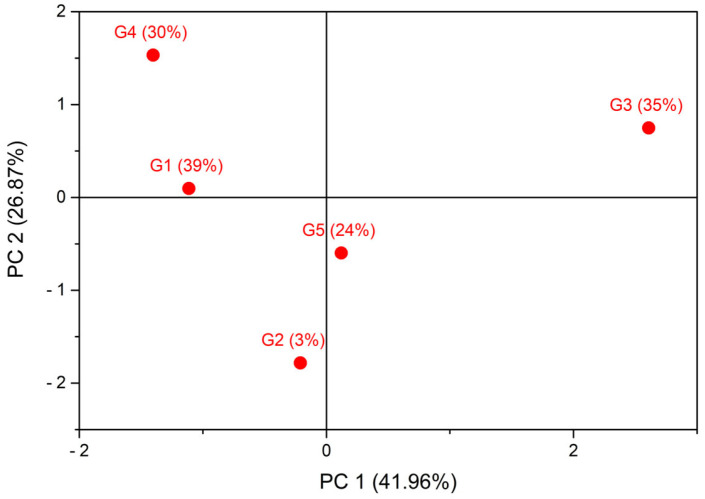
Score plot of the exploratory principal component analysis (PCA) of post-harvest traits in lettuce under five greenhouse UV-B transmittances. Each point represents a greenhouse treatment (G), labeled with its UV-B transmittance (%). PC1 and PC2 explain 41.96% and 26.87% of the total variance, respectively. The plot illustrates coordinated, non-linear responses of yield and nutritional traits along the UV-B gradient. Correlation matrices and variable loadings are reported in Supplementary Tables S16 and S17.

A preliminary, exploratory principal component analysis (PCA), as conducted for eggplant, was applied to post-harvest variables to provide an integrated overview of coordinated responses across the UV-B gradient. The PCA score plot (Figure 7) shows a structured separation of the five UV-B treatments, indicating coordinated but non-linear trait responses.

Correlation matrices and variable loadings are reported Tables S16 and S17, providing additional insights into the relationships among post-harvest traits. The first two components explained 41.96% and 26.87% of the total variance, respectively, for a cumulative variance of 68.8%. The total variance explained is lower than that observed for eggplant (85.8%), suggesting a more heterogeneous response of post-harvest traits under UV-B exposure and the presence of variables with weaker intercorrelations, which disperse the variance across additional components. PC1 primarily captured variation associated with antioxidant activities (HAA and LAA) and yield, while PC2 was mainly related to Vitamin C and chlorophyll a + b.

Overall, these exploratory analyses indicate that moderate UV-B exposure optimizes both productivity and nutritional quality in lettuce, while the highest UV-B levels primarily enhance antioxidant and vitamin C content without further yield gains.

### 2.5. Comparative Analysis of Crop Responses and Optimal UV-B Transmittance

A comprehensive comparison of eggplant and lettuce under greenhouse covers with different UV-B transmittances revealed clear species-specific responses across thermal, physiological, yield, and nutritional parameters.

**Figure 8:**
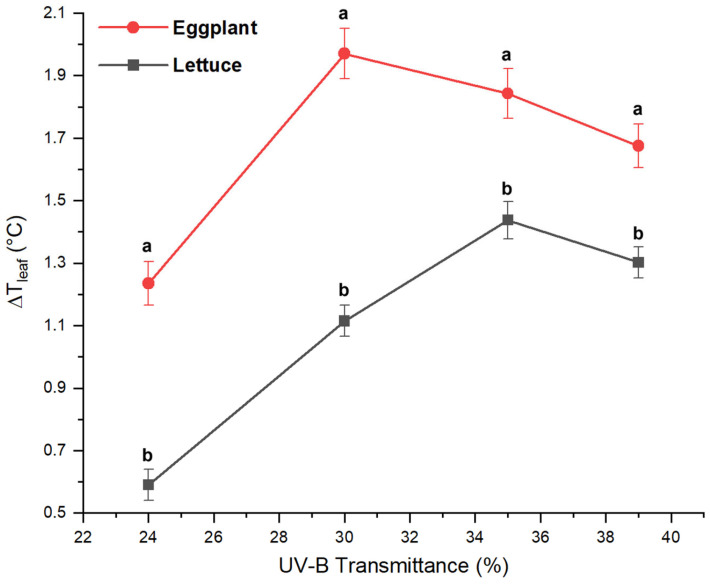
Comparison of leaf temperature differences (Δ*T_leaf_*) between eggplant and lettuce. Average Δ*T_leaf_* at four growth stages (10, 24, 38, 52 DAT) under greenhouses with varying UV-B transmittances (3%, 24%, 30%, 35%, 39%). Values represent mean ± SD for each treatment. Different letters indicate statistically significant differences between the two cultivars within each UV-B treatment (*p* < 0.05).

Leaf temperature differences (Δ*T_leaf_*) highlighted distinct thermal sensitivities between the two species (Figure 8). On average, eggplant exhibited higher Δ*T_leaf_* than lettuce at all UV-B levels, consistent with its larger leaf area, thicker leaves, and altered transpiration dynamics. Both species, however, shared a common response pattern: Δ*T_leaf_* increased from low to intermediate UV-B transmittances and then stabilized or slightly decreased at the highest transmittance (39%). Lettuce reached maximal Δ*T_leaf_* under 35% UV-B, whereas eggplant peaked at 30%, indicating species-specific thermal regulation strategies. Importantly, leaf temperature differences should not be interpreted as a direct thermal effect of UV-B radiation. Rather, Δ*T_leaf_* reflects UV-B-induced modifications in plant physiological regulation, including changes in stomatal conductance, transpiration rates, and the accumulation of UV-screening secondary metabolites [51,52,53]. The observed saturation and partial decline of Δ*T_leaf_* at the highest UV-B transmittance suggest that both crops activate effective protective mechanisms that limit excessive leaf warming and prevent thermal stress. Similar thermal dynamics have been reported under controlled UV-B irradiation [54], showing progressive leaf warming up to a threshold, beyond which compensatory mechanisms mitigate additional temperature increases. Although the absolute UV-B doses and sources differ between natural and artificial conditions, these findings support the concept that plants regulate leaf energy balance through integrated physiological and biochemical responses to UV-B exposure, confirming the relevance of Δ*T_leaf_* as an indicator of plant acclimation.

**Figure 9:**
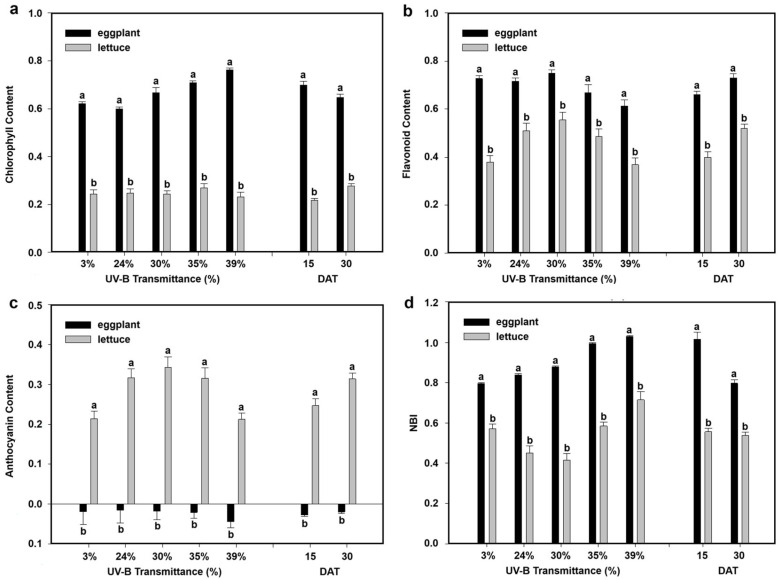
Comparative physiological responses of eggplant and lettuce under varying UV-B transmittances. Four bar charts report: (**a**) chlorophyll content, (**b**) flavonoid accumulation, (**c**) anthocyanin content, and (**d**) nitrogen index (IN). Different letters indicate statistically significant differences between the two cultivars within each UV-B treatment (*p* < 0.05). Bars represent mean values at 15 and 30 DAT for each UV-B treatment.

Physiological measurements revealed complementary patterns across the two species. In Figure 9 the physiological data obtained for eggplant and lettuce are reported side by side to facilitate direct comparison. Eggplant exhibited higher chlorophyll content across all UV-B conditions, confirming its greater photosynthetic robustness under enhanced UV-B exposure. In contrast, lettuce showed relatively stable chlorophyll levels, with variations mainly driven by plant age rather than UV-B intensity. Flavonoid and anthocyanin accumulation further emphasized distinct acclimation strategies. Lettuce displayed pronounced increases at intermediate UV-B transmittances, indicating a strong reliance on UV-screening secondary metabolites as a primary defense mechanism. Eggplant, by contrast, showed a more moderate and less UV-B-dependent response, suggesting a reduced dependence on flavonoid- and anthocyanin-based screening and a greater contribution of structural or metabolic adjustments.

Similarly, the NBI exhibited contrasting behaviors. Eggplant showed a strong increase with increasing UV-B transmittance, particularly at early growth stages, whereas lettuce displayed a more variable response with maximum values at the highest UV-B level but limited temporal variation. These differences likely reflect divergent nitrogen allocation strategies, with eggplant investing more heavily in nitrogen-rich compounds supporting sustained growth and stress tolerance under elevated UV-B radiation.

These physiological patterns were consistent with the observed thermal trends. In eggplant, increased Δ*T_leaf_* under intermediate UV-B conditions was associated with higher chlorophyll content and nitrogen index, suggesting intensified metabolic activity and altered transpiration dynamics. In lettuce, the highest Δ*T_leaf_* values under intermediate UV-B transmittances corresponded to conditions promoting enhanced flavonoid and anthocyanin accumulation, indicating active modulation of photoprotective pathways. At the highest UV-B transmittance (39%), both species exhibited stabilization or slight reductions in physiological parameters, consistent with the observed attenuation of Δ*T_leaf_*, suggesting dose-dependent regulatory mechanisms that balance photoprotection, metabolic activity, and thermal regulation under elevated UV-B exposure.

Yield and nutritional traits further confirmed species-specific responses. Lettuce productivity and metabolic activity were maximized under intermediate UV-B (30–35%), with enhanced accumulation of flavonoids, anthocyanins, and nitrogen-rich compounds. Eggplant, in contrast, required higher UV-B (35–39%) to achieve peak nutraceutical content, particularly in antioxidant capacity and vitamin C, while yield remained relatively stable across treatments.

In addition to species-specific traits, the seasonal context likely contributed to the observed differences between eggplant (May–July) and lettuce (October–December). The radiometric and micromilatic data reported in Tables S4–S11 indicate that eggplant was cultivated under higher ambient air temperatures, higher PAR levels, and generally higher vapor pressure deficit (VPD) compared to lettuce, which developed under cooler autumn–winter conditions with lower incoming radiation.

Under the spring–summer cycle, the combination of elevated UV-B, higher PAR, and increased VPD likely enhanced transpiration demand and leaf energy load in eggplant, contributing to the stronger Δ*T_leaf_* responses observed at intermediate UV-B transmittances. In contrast, lettuce grown during autumn–winter experienced lower thermal and radiative pressure, which may have favored the activation of photoprotective pathways (flavonoids and anthocyanins) at intermediate UV-B levels without inducing pronounced thermal stress.

The observed increases in antioxidant activities and vitamin C content under higher UV-B transmittances are consistent with UV-B-specific signaling mechanisms. UV-B is perceived by the photoreceptor UVR8, which undergoes conformational changes upon irradiation and initiates a signaling cascade that activates downstream transcription factors such as HY5 [55,56]. These transcription factors regulate the expression of key genes in the phenylpropanoid and flavonoid biosynthetic pathways, promoting the accumulation of UV-screening and antioxidant secondary metabolites [56,57,58,59]. Consequently, the elevated flavonoid, anthocyanin, and vitamin C levels observed in both lettuce and eggplant likely reflect this UVR8-mediated metabolic reprogramming, which also contributes to enhanced photoprotection.

Overall, the integration of thermal, physiological, yield, and nutritional data indicates that UV-B transmittances of 30–35% represent apparent optima for lettuce under these experimental conditions, promoting active photoprotection and secondary metabolite accumulation without inducing thermal stress. In eggplant, higher UV-B transmittances of 35–39% is necessary to elicit maximal metabolic and nutraceutical responses, consistent with its thicker leaves and greater tolerance to elevated UV-B.

These differences in optimal ranges likely reflect intrinsic differences in leaf morphology, growth rate, and photoprotective capacity between the two species [60,61,62]. Leafy crops such as lettuce, with high surface-to-volume ratio and rapid growth, typically respond strongly to intermediate UV-B doses that stimulate secondary metabolite accumulation and photosynthetic activity without inducing thermal stress. Fruit-bearing species with thicker leaves and slower growth, such as eggplant, generally require higher UV-B exposure to elicit comparable metabolic and protective responses [62,63,64,65]. However, Seasonal differences in background radiation and microclimate may therefore have modulated the physiological sensitivity to UV-B, influencing both the magnitude of Δ*T_leaf_* variation and the balance between growth and secondary metabolism. These findings suggest that the identified apparent optimal UV-B transmittance ranges should be interpreted within the specific seasonal context of each crop cycle, as plant responses likely result from the interaction between UV-B modulation and baseline environmental conditions.

These results are in line with previous greenhouse studies reporting enhanced flavonoid accumulation and antioxidant capacity in lettuce cultivated under UV-transmitting films compared with UV-blocking covers [17,23,24,25,26,27]. Earlier investigations demonstrated that even moderate natural UV-B exposure can stimulate secondary metabolism without impairing growth performance. Our findings refine these observations by quantitatively identifying intermediate UV-B transmittance (30–35%) as an apparent optimal range for lettuce under Mediterranean greenhouse conditions.

Similarly, the enhanced antioxidant activity and vitamin C concentration observed in eggplant at higher UV-B levels (35–39%) corroborate the recognized role of UV-B as a natural elicitor of phenylpropanoid metabolism, as demonstrated in other horticultural crops [17,18,19,20,21,22].

However, most previous studies focused on single species or short-term UV-B exposure, whereas the present work provides a comparative multi-species assessment across a broad UV-B gradient.

It should be noted that, the plastic films also induced variations in UV-A radiation (Table S2), ranging from approximately 54–59%. Nevertheless, this variation was considerably smaller and physiologically less pronounced than the differences observed in UV-B transmittance, which ranged from 3% to 39%. Furthermore, UV-A transmittance did not follow the same gradient as UV-B among the films, suggesting that the observed shifts in thermal, physiological, and nutraceutical traits were largely associated with UV-B modulation, while potential UV-A effects may have contributed as secondary factors.

Each UV-B transmittance level was applied at single greenhouse and evaluated during a single growing season, which restricts formal post hoc power analysis. Nevertheless, the controlled greenhouse environment, repeated measurements across growth stages, and combined assessment of thermal, physiological, and nutritional traits provide robust comparative insights into species-specific responses to UV-B modulation.

Overall, these results indicate that modulation of UV-B transmittance in greenhouse coverings can drive coordinated changes in plant physiological regulation and functional quality. However, to confirm the consistency of these responses and enhance inferential power and generalizability, future studies incorporating multi-season trials, film rotation among greenhouses, and multi-site replication are recommended.

## 3. Materials and Methods

### 3.1. Plastic Films and UV–VIS Spectral Characterization

Commercial plastic films were used to cover the experimental greenhouses. Four films (G1, G3, G4, and G5) featured a UV-B–transmitting window, while a control film was designed to block most ultraviolet radiation. The UV-B–transmitting films were supplied by Ginegar Plastic Products Ltd. (Kibbutz Ginegar, Israel), and the UV-B–blocking film was provided by Lirsa SpA (Ottaviano (NA), Italy). Different levels of UV-B transmission in the Ginegar films were achieved by varying the content of UV-absorbing additives incorporated into the plastics. All five films had a similar thickness of 180 µm and shared key characteristics, including diffused light transmission, high thermal stability, and an anti-drip system.

Spectral characterization of the greenhouse films was carried out using a UV–VIS spectrophotometer (JASCO V-650; spectral accuracy ±0.2 nm, wavelength range 190–850 nm). Total transmittance spectra, *T(λ)*, were measured using an integrating sphere (JASCO ISN-722, inner diameter 60 mm, operating range 200–870 nm), which accounts for both direct and diffuse components of transmitted radiation. The integrating sphere was calibrated according to the manufacturer’s standard procedure using the provided reference standards prior to measurements to ensure spectral accuracy.

Average spectral transmittance $T_{av}\;$was calculated from the measured spectra over selected wavelength intervals using the following expression:

(1) $$T_{av}=\frac{\int_{a}^{b}T\left(\lambda \right)d\lambda }{b-a}$$

where the integration limits (*a*,*b*) were set to 280–315 nm for UV-B, 315–400 nm for UV-A, and 400–700 nm for PAR. $T_{av}$ calculated for UV-B, UV-A, and PAR for the five greenhouse films, are reported in Table S1.

### 3.2. Experimental Design, Plant Materials, and Crop Management

The experiment was conducted during the 2024 growing season in the Acerra area (Naples, Campania Region, Italy) at the private experimental farm ARCA 2010 SCARL (latitude: 40°57′56.65″ N, longitude: 14°25′41.24″ E, altitude: ~60 m a.s.l.). The experimental setup consisted of five greenhouses, each 10.00 m long and 5.80 m wide, covering a total area of 58 m^2^, with a 2 m spacing between them. The plastic films were randomly assigned to the five greenhouses according to their measured UV-B transmittance. Each greenhouse structure represented the experimental unit. All greenhouses were located within the same experimental facility and were managed under identical agronomic and irrigation practices to minimize environmental heterogeneity.

Although each plastic film was applied to a single greenhouse, the controlled environment minimized environmental variability. Multiple plants were monitored per greenhouse, and repeated measurements at multiple growth stages allowed comparative assessment of treatment effects.

Two crop species were grown under this system: eggplant (*Solanum melongena* L., cv. Lunga Napoletana, a traditional long-shaped cultivar originating from southern Italy) and lettuce (*Lactuca sativa* L., cv. Rosplus). The eggplant cultivar ‘Lunga Napoletana’ was selected due to its wide diffusion in Mediterranean greenhouse production systems and its well-characterized agronomic performance under protected cultivation, making it representative of commercially relevant genotypes in Southern Europe. The lettuce cultivar ‘Rosplus’, a red-leaf type characterized by active phenylpropanoid metabolism and potential for flavonoid and anthocyanin accumulation, was chosen to allow evaluation of UV-B–induced modulation of secondary metabolites in leafy vegetables with high nutritional value. The inclusion of two botanically and morphologically distinct species (a fruit-bearing Solanaceae and a leafy Asteraceae) enables a comparative assessment of species-specific responses to UV-B modulation. Although the results strictly refer to the selected cultivars, both are agronomically representative of their respective crop categories, thereby supporting cautious generalization of physiological trends to similar greenhouse-grown genotypes under Mediterranean conditions.

Eggplants were transplanted on 15 April 2024. The planting density was 1.2 m × 0.4 m, corresponding to 2.08 plants m^−2^, with 25 plants per row, each corresponding to 1 replica (total 3 replicates), for a total of 75 plants per module, and an overall total of 375 plants. Lettuce was transplanted on 24 October 2024 at a spacing of 0.30 m × 0.35 m, corresponding to 9.52 plants m^−2^, with 27 plants per each of 6 rows, corresponding to six replicates, for a total of 162 plants per module, and a general amount of 810 plants.

Both crops were cultivated following standard local agronomic practices for soil preparation, and fertilization. Irrigation was applied daily to maintain optimal soil moisture, and pest management followed integrated crop protection guidelines. Harvesting was carried out on 17 July 2024 for eggplants and on 18 December 2024 for lettuce under the five greenhouses.

### 3.3. Infrared Thermography (IT) Measurements

IT measurements were carried out using an LWIR AVIO TVS500 camera (Nippon Avionics Co., Yokohama, Japan) equipped with an uncooled microbolometer detector (spectral range 8–14 µm, FPA 320 × 240 pixels, NETD ~50 mK at 25 °C) and a 22 mm focal lens (IFOV 1.68 mrad). Temperature monitoring and basic image processing were performed in real-time using the commercial software IRT Analyzer ver. 4.8 (GRAYESS Inc., Bradenton, FL, USA) supplied with the camera. The mean emissivity values were experimentally determined using a calibrated black reference disc. The resulting emissivity was 0.95 for eggplant, and 0.96 for lettuce. These values were considered into the thermal data processing to ensure accurate temperature estimation.

For both crops, measurements were carried out at four growth stages: 10, 24, 38, and 52 Days After Transplanting (DAT). In each DAT, measurements were taken within the same time window (10:00–12:00 a.m.). During each session, 40 thermal images of the leaf canopy were captured per crop under each greenhouse, focusing on plants located in the central area and excluding the first and last 3 m of each plot to avoid edge effects. The mean leaf temperature for each greenhouse was estimated from at least three leaves per image. Subsequently, the average leaf temperature difference (Δ*T_leaf_*) was calculated for the crops under greenhouses with UV-B transmittances of 24%, 30%, 35%, and 39% using the following equation:

(2) $$\Delta T_{leaf}=T_{m}-T_{m,ref}$$

where $T_{m}$ is the mean leaf temperature for a given UV-B treatment, and $T_{m,ref}$ is the mean leaf temperature measured under the reference greenhouse (3% UV-B).

IT was selected as a non-invasive technique to monitor plant physiological responses at canopy scale throughout the growth cycle. Leaf temperature represents an integrated indicator of plant energy balance, influenced by stomatal conductance, transpiration rate, radiation absorption, and metabolic activity. Because stomatal regulation directly affects both transpiration and photosynthetic carbon assimilation, variations in leaf temperature are closely associated with changes in stomatal conductance and photosynthetic performance [37,45,52,66]. The theoretical and experimental basis linking infrared thermometry to stomatal behavior and canopy physiological regulation has been extensively documented in previous studies [52,66].

Although IT does not provide direct measurements of net photosynthetic rate, it allows rapid, repeated, and spatially extensive assessment of plant physiological regulation under field or greenhouse conditions, without disturbing the crop. Compared to point-based gas exchange measurements, which are time-consuming and limited to individual leaves, thermal imaging enables monitoring of whole-canopy responses under realistic agronomic conditions [30,31].

IT should therefore be regarded as a proxy indicator rather than a direct measurement of photosynthesis. While it does not quantify net CO_2_ exchange, its sensitivity to changes in stomatal behavior and leaf energy balance makes it particularly suitable for comparative analyses of plant physiological responses under different UV-B transmittance regimes.

### 3.4. Radiation and Microclimate Monitoring

For both crops (eggplant and lettuce), UV-B radiation and PAR were measured inside and outside the experimental greenhouses on the same days corresponding to thermographic assessments (DAT 10, 24, 38, and 52).

On each monitoring day, 24 measurements were recorded between 10:00 and 12:00, assessing radiation levels outside the greenhouse and inside each greenhouse at canopy height, in order to characterize the effective radiation environment experienced by the plants under each plastic film. Measurements were performed using a photoradiometer with datalogger (HD 2102.2, Delta Strumenti S.r.l., Varese, Italy) equipped with a UV probe (LP 471 UVB) for UV-B detection and a PAR probe (LP 471 PAR). Mean values (±SD) of UV-B and PAR are reported in Tables S2 and S3.

On the same days and within the same time interval (10:00–12:00), air temperature (*T*, °C) and relative humidity (*RH*, %) were recorded inside each greenhouse using a thermo-hygrometer (RS PRO, RS Group plc, London, UK). Based on these measurements, vapor pressure deficit (*VPD*) was calculated according to the following equation:

(3) $$VPD=e_{s}(T)\times \left(1-\frac{RH}{100}\right)$$

where $e_{s}(T)$ represents the saturation vapor pressure (kPa), calculated using Tetens’ equation with air temperature (T, °C) [67,68]:

(4) $$e_{s}(T)=0.6108\cdot exp\left(\frac{17.27\cdot T}{T+237.3}\right)$$

Mean values (±SD) of air temperature, relative humidity, and VPD for both crops are reported in Tables S6–S11.

### 3.5. Physiological Measurements

In vivo physiological measurements were conducted using a Multi-Pigment Meter (MPM-100, ADC Bioscience Ltd., Hoddesdon, UK) to estimate chlorophyll, flavonoid, and anthocyanin content, as well as the nitrogen–flavonol index (NFI). This non-destructive optical device is widely used to assess leaf-level physiological parameters [69]. Previous studies have shown that such optical measurements reliably correlate with standard biochemical assays for pigment quantification [70,71,72]. These approaches are particularly suitable for comparative analyses of treatment effects, as they effectively capture relative changes in pigment concentrations across leaves and experimental units.

The device calculates chlorophyll content based on the ratio of leaf transmission in the far-red (850 nm) and near-infrared (720 nm) regions. Flavonoid content is determined from the ratio of fluorescence emission in the red region (660 nm) to fluorescence in the UV-A region (325 nm), while anthocyanin content is measured from the ratio of red (660 nm) to green (525 nm) fluorescence. The nitrogen–flavonol index (NFI) is estimated as the ratio between the measured chlorophyll and flavonoid contents.

Measurements were carried out on fully expanded leaves of both crops at two growth stages: 15 and 30 Days After Transplanting (DAT). For each greenhouse, 20 randomly selected plants per crop were measured, avoiding edge rows to minimize microclimatic effects. The average values for each parameter and treatment were calculated from all measured leaves for comparative analysis across UV-B treatments.

### 3.6. Post-Harvest Nutritional Analyses

At harvest, the above ground biomass of lettuce and the fruits of eggplant were collected from each greenhouse to assess yield, expressed as tons per hectare. Samples for the different analyses were taken from three separate greenhouse locations to evaluate possible differences in plant responses. Then, five representative fresh samples of lettuce leaves and eggplant fruits were immediately frozen at −80 °C, successively lyophilized (Alpha 1-4 LSCplus, Christ, Osterode, Germany) in order to determine: Hydrophilic antioxidant activity (HAA); Lipophilic antioxidant activity (LAA); total carotenoids and total chlorophyll (Chl a + b).

The HAA, expressed as mmol ascorbic acid per 100 g of dry weight (dw), was assessed spectrophotometrically, after adding distilled water, according to the method of Fogliano et al. [73]; the absorbance of hydrophilic extract fractions was measured at 505 nm. The LAA was determined on 200 mg of freeze-dried material using methanol through a spectrophotometer (Hach DR 2000, Hach Co., Loveland, CO, USA) according to the methods of Re et al. [74]; the absorbance of solution was measured at 734 nm and the results were expressed as mmol of Trolox per 100 g of dry weight (dw). Chlorophyll and carotenoid content was also measured spectrophotometrically after the extraction with ammoniacal acetone according to the method described by Wellburn [75]. Solution absorbances were assessed at 662 and 647 for chlorophyll a and b, at 470 nm for carotenoids. Total chlorophyll (chlorophyll a and b), and carotenoids were expressed as µg mL^−1^ and mg 100 g^−1^ fw, respectively. Finally, Vitamin C (total ascorbic acid, expressed as mg 100 g^−1^ fresh weight) was determined spectrophotometrically on fresh material according to the protocol stated by Kampfenkel et al. [76] and the solution absorbance was measured at 525 nm.

### 3.7. Statistical Analysis

Productive and post-harvest data were analyzed by one-way ANOVA using SPSS (v.22, IBM, Chicago, IL, USA), with mean comparisons performed via Tukey’s test at *p* < 0.01 and *p* < 0.05. Data are reported as mean ± standard deviation. Leaf temperature data were analyzed in two ways: (i) to evaluate temporal changes, a one-way repeated measures ANOVA was performed separately for each cultivar under each UV-B treatment, with Tukey’s post hoc test used to identify statistically significant differences among the four sampling times (10, 24, 38, and 52 DAT, *p* < 0.05); (ii) to compare cultivars, a one-way ANOVA was performed for each UV-B treatment, with Tukey’s test used to detect significant differences between the two cultivars (*p* < 0.05). In addition, multivariate relationships among post-harvest traits were explored using principal component analyses (PCA) performed in Origin 2025 (OriginLab, Northampton, MA, USA).

## 4. Conclusions

This study quantitatively demonstrates that greenhouse UV-B transmittance between 30% and 39% of ambient radiation differentially modulates productivity and nutraceutical quality in eggplant (*Solanum melongena* L., cv. Lunga Napoletana) and lettuce (*Lactuca sativa* L., cv. Rosplus). Lettuce achieved maximum yield and enhanced accumulation of flavonoids, anthocyanins, and chlorophyll under intermediate UV-B levels (30–35%), with post-harvest increases in total antioxidant activity and vitamin C content of up to 204% compared with the lowest UV-B transmittance treatment (reference greenhouse).

Conversely, eggplant reached peak antioxidant activity and vitamin C concentrations at higher UV-B transmittance (35–39%), showing increases of approximately ~132% and ~45%, respectively, without significant yield penalties. Carotenoid and total chlorophyll contents were similarly enhanced under elevated UV-B, confirming the stimulatory effect of moderate-to-high UV-B exposure on secondary metabolism.

At the highest UV-B level (39%), leaf temperature variations remained limited (≈1 °C range across DAT), and physiological parameters showed stabilization or slight reductions, indicating the activation of dose-dependent regulatory mechanisms that preserve photoprotection and metabolic balance rather than inducing stress-related impairment.

These findings indicate that the effects of UV-B are species-dependent: in lettuce, intermediate UV-B levels improve both productivity and phytochemical content, while in eggplant, higher UV-B enhances nutraceutical quality without compromising yield.

From an applied perspective, selecting greenhouse films with tailored UV-B transmittance (≈30–35% for lettuce; ≈35–39% for eggplant) represents a practical and technically feasible strategy to improve functional quality without compromising agronomic performance under Mediterranean greenhouse conditions. However, the reported quantitative responses reflect the specific environmental conditions, cultivars, and experimental setup adopted in this study and should therefore be interpreted within the tested Mediterranean context rather than as universally transferable thresholds.

## Figures and Tables

**Table 1:** Yield and post-harvest nutritional traits of eggplant grown under greenhouse covers with different UV-B transmittances. Values are expressed as mean ± standard deviation (SD).

%UV B	Yield (t ha^−1^)	HAA (mmol Ascorbic Acid equ 100 g^−1^ dw)	LAA (mmol Trolox equ 100 g^−1^ dw)	Vitamin C (mg 100 g^−1^ fw)	Carotenoids (mg 100 g^−1^ fw)	Chlor a + b (µg mL^−1^)
3	85.8 ± 0.3	3.32 ± 0.28 b	7.5 ± 0.5 d	44.5 ± 1.5 bc	0.014 ± 0.001 a	0.120 ± 0.005
24	77.0 ± 0.3	5.44 ± 0.39 a	11.1 ± 0.6 cd	40.3 ± 5.2 c	0.006 ± 0.001 b	0.110 ± 0.009
30	73.7 ± 0.3	5.38 ± 0.13 a	16.8 ± 0.8 ab	41.9 ± 0.9 bc	0.003 ± 0.001 b	0.130 ± 0.005
35	81.3 ± 0.3	4.67 ± 0.49 ab	17.4 ± 1.3 a	60.8 ± 2.8 ab	0.005 ± 0.001 b	0.130 ± 0.002
39	85.0 ± 0.3	5.91 ± 0.43 a	13.3 ± 0.7 bc	64.6 ± 7.2 a	0.007 ± 0.001 b	0.130 ± 0.012
Significance	ns	*	*	*	*	ns

* indicates significance at *p* < 0.01. In each column, different letters indicate significant differences.

**Table 2:** Yield and post-harvest nutritional traits of lettuce grown under greenhouse covers with different UV-B transmittances. Values are expressed as mean ± standard deviation (SD).

%UV B	Yield (t ha^−1^)	HAA (mmol Ascorbic Acid equ 100 g^−1^ dw)	LAA (mmol Trolox equ 100 g^−1^ dw)	Vitamin C (mg 100 g^−1^ fw)	Carotenoids (mg 100 g^−1^ fw)	Chlor a + b (µg mL^−1^)
3	30.2 ± 1.6 b	5.02 ± 0.50 b	3.90 ± 0.43 b	13.4 ± 1.0 c	0.18 ± 0.01 b	0.63 ± 0.12 ab
24	40.4 ± 0.9 a	5.33 ± 0.35 b	3.20 ± 0.55 b	16.9 ± 3.7 bc	0.25 ± 0.05 ab	0.39 ± 0.06 b
30	38.5 ± 0.7 a	5.29 ± 0.37 b	3.16 ± 0.54 b	20.3 ± 1.3 b	0.36 ± 0.01 a	0.98 ± 0.04 a
35	40.8 ± 0.9 a	7.23 ± 0.03 a	7.00 ± 0.67 a	15.3 ± 1.8 bc	0.20 ± 0.05 ab	0.66 ± 0.11 ab
39	34.2 ± 2.3 ab	5.08 ± 0.26 b	4.68 ± 0.69 ab	40.7 ± 3.4 a	0.29 ± 0.04 ab	0.65 ± 0.04 ab
Significance	*	*	*	*	**	*

* and ** indicate significance at *p* < 0.01 and *p* < 0.05, respectively. In each column, different letters indicate significant differences.

## Data Availability

The raw data supporting the conclusions of this article will be made available by the authors on request. The data are not publicly available mainly due to its size and quantity (numerous infrared images).

## Supplementary Materials

The following supporting information can be downloaded at: https://www.mdpi.com/article/10.3390/plants15060863/s1.
