# Supplementary material for: Yield, Nutritional, and Thermal Responses of Lettuce (Lactuca sativa) and Eggplant (Solanum melongena) Under Greenhouse Covers with Different UV-B Transmittance
Source: Plants (Basel). 2026 Mar 11;15(6):863. doi: 10.3390/plants15060863 (PMC13030510; doi:10.3390/plants15060863)
Supplement: Supplementary file 1 [file plants-15-00863-s001.zip › Supplementary File.pdf]

Supplementary Data

Yield, Nutritional, and Thermal Responses of Lettuce (*Lactuca sativa*) and Eggplant (*Solanum melongena*) under Greenhouse Covers with Different UV-B Transmittance

Mauro Mori[1], Eugenio Cozzolino[2], Ida Di Mola[1], Lucia Ottaiano[1], Antimo Di Meo[3], Pasquale Mormile[3], Massimo Rippa[3,*]

[1] Department of Agricultural Sciences, University of Naples Federico II, 80055 Portici, Naples, Italy; mori@unina.it (M.M); ida.dimola@unina.it (I.D.M.); lucia.ottaiano@unina.it (L.O.)
[2] Council for Agricultural Research and Economics (CREA)—Research Center for Cereal and Industrial Crops, 81100 Caserta, Italy; eugenio.cozzolino@crea.gov.it (E.C.)
[3] Institute of Applied Sciences and Intelligent Systems "E. Caianiello" of National Research Council of Italy (CNR ISASI), Via Campi Flegrei 34, 80072 Pozzuoli, Naples, Italy; a.dimeo@isasi.cnr.it (A.D.M.); p.mormile@isasi.cnr (P.M.)
* Correspondence: m.rippa@isasi.cnr.it

1. Spectral transmittance of the greenhouse films

Table S1. Average spectral transmittance (%) of UV-B (280–315 nm), UV-A (315–400 nm), and photosynthetically active radiation (PAR, 400–700 nm) for the five greenhouse films, measured in the laboratory using UV–VIS spectrophotometry. Values are reported as mean ± standard deviation (SD).

	G1	G2	G3	G4	G5
UV-B (%)	38.9 ± 0.3	3.2 ± 0.2	34.8 ± 0.3	29.8 ± 0.3	24.3 ± 0.2
UV-A (%)	56.0 ± 0.3	54.2 ± 0.3	57.5 ± 0.4	58.8 ± 0.3	55.0 ± 0.3
PAR (%)	85.0 ± 0.4	85.8 ± 0.4	84.7 ± 0.4	84.7 ± 0.3	84.0 ± 0.3

2. Average leaf temperatures

Table S2. Average leaf temperatures (T_{leaf}, °C) measured by infrared thermography in eggplant canopies under five greenhouse covers (G1-5) with different UV-B transmittances during the two-hour period 10:00–12:00 on four Days After Transplanting (DAT 10, 24, 38, and 52). Values represent mean ± standard deviation (SD).

DAT	T_{leaf} (°C)				
	G1	G2	G3	G4	G5
10	26.35 ± 0.04	24.83 ± 0.03	26.36 ± 0.04	26.54 ± 0.04	25.94 ± 0.03
24	29.83 ± 0.04	28.22 ± 0.03	29.95 ± 0.05	30.15 ± 0.05	29.38 ± 0.03
38	32.88 ± 0.05	31.12 ± 0.03	33.06 ± 0.05	33.15 ± 0.05	32.36 ± 0.04
52	34.13 ± 0.05	32.32 ± 0.03	34.49 ± 0.05	34.53 ± 0.05	33.75 ± 0.04

Table S3. Average leaf temperatures (T_{leaf} , °C) measured by infrared thermography in lettuce canopies under five greenhouse covers (G1-5) with different UV-B transmittances during the two-hour period 10:00–12:00 on four Days After Transplanting (DAT 10, 24, 38, and 52). Values represent mean ± standard deviation (SD).

DAT	T_{leaf} (°C)				
	G1	G2	G3	G4	G5
10	22.66 ± 0.03	21.21 ± 0.03	22.85 ± 0.03	22.44 ± 0.03	21.85 ± 0.03
24	19.60 ± 0.02	18.22 ± 0.03	19.79 ± 0.03	19.40 ± 0.02	18.82 ± 0.02
38	14.68 ± 0.02	13.41 ± 0.02	14.82 ± 0.02	14.52 ± 0.02	13.98 ± 0.02
52	12.84 ± 0.03	11.73 ± 0.02	12.86 ± 0.02	12.67 ± 0.02	12.28 ± 0.02

3. Radiation and Microclimate measurements

Table S4. Average UV-B radiation values (mW m^{-2}) recorded outside and inside the five experimental greenhouses at canopy height over a 2-hour monitoring period (10:00–12:00) on the four days corresponding to thermal imaging assessments (DAT 10, 24, 38, and 52) for eggplant and lettuce. Values are expressed as mean ± standard deviation (SD).

Cultivation	DAT	UV-B (mW/m^2)					
		Outside	G1	G2	G3	G4	G5
Eggplant	10	271 ± 6	105 ± 3	8 ± 1	94 ± 3	81 ± 3	66 ± 3
	24	312 ± 7	122 ± 4	10 ± 1	109 ± 4	94 ± 3	75 ± 3
	38	382 ± 7	148± 4	13 ± 1	134 ± 4	113 ± 4	92 ± 4
	52	361 ± 7	141 ± 4	11 ± 1	125 ± 4	107 ± 3	87 ± 4
Lettuce	10	120 ± 3	47 ± 2	3.7± 0.5	42 ± 2	36 ± 3	29 ± 2
	24	86 ± 3	33 ± 2	2.8 ± 0.4	30 ± 2	25 ± 3	21 ± 2
	38	75 ± 2	29 ± 2	2.2 ± 0.4	26 ± 2	22 ± 2	18 ± 2
	52	49 ± 2	19 ± 2	1.6 ± 0.3	17 ± 1	15 ± 2	12 ± 2

Table S5. Average photosynthetically active radiation (PAR) values (μmol m^{-2} s^{-1}) recorded outside and inside the five experimental greenhouses at canopy height over a 2-hour monitoring period (10:00–12:00) on the four days corresponding to thermal imaging assessments (DAT 10, 24, 38, and 52) for eggplant and lettuce. Values are expressed as mean ± standard deviation (SD).

Cultivation	DAT	PAR (μmol m^{-2} s^{-1})					
		Outside	G1	G2	G3	G4	G5
Eggplant	10	1325 ± 32	1127 ± 33	1139 ± 34	1126 ± 35	1122 ± 33	1110 ± 32
	24	1377 ± 35	1166 ± 34	1187 ± 35	1161 ± 34	1170 ± 34	1160 ± 34
	38	1421 ± 35	1212 ± 36	1218 ± 36	1206 ± 36	1200 ± 36	1189 ± 35
	52	1380 ± 36	1170 ± 35	1180 ± 34	1166 ± 35	1169 ± 35	1157 ± 35
Lettuce	10	910 ± 21	771 ± 18	780 ± 18	772 ± 18	776 ± 18	767 ± 18
	24	724 ± 23	618 ± 17	623 ± 17	609 ± 17	615 ± 17	607 ± 16
	38	620 ± 20	529 ± 15	535 ± 15	525 ± 15	522 ± 15	520 ± 15
	52	582 ± 20	492 ± 15	497 ± 15	494 ± 15	492 ± 15	487 ± 15

Table S6. Average air temperature (T, °C) recorded inside the five experimental greenhouses for eggplant at four key growth stages (DAT 10, 24, 38, and 52). Values represent the mean ± standard deviation (SD).

DAT	T (°C)				
	G1	G2	G3	G4	G5
10	25.2 ± 0.6	24.6 ± 0.5	25.1± 0.6	24.7 ± 0.5	24.5 ± 0.5
24	27.5 ± 0.6	27.8 ± 0.6	27.2 ± 0.6	26.9 ± 0.5	26.7 ± 0.6
38	30.2 ± 0.7	30.6 ± 0.7	29.7 ± 0.6	30.3 ± 0.7	29.8 ± 0.6
52	31.3 ± 0.7	31.6 ± 0.7	31.0 ± 0.6	31.3 ± 0.6	31.0 ± 0.6

Table S7. Average relative humidity (RH, %) recorded inside the five experimental greenhouses for eggplant at four key growth stages (DAT 10, 24, 38, and 52). Values represent the mean ± standard deviation (SD).

DAT	RH (%)				
	G1	G2	G3	G4	G5
10	56 ± 3	60 ± 4	57 ± 3	57 ± 3	58 ± 4
24	54 ± 3	55 ± 4	56 ± 4	55 ± 3	57 ± 3
38	51 ± 4	54 ± 3	55 ± 4	52 ± 4	54 ± 3
52	50 ± 3	50 ± 3	51 ± 3	51 ± 4	53 ± 3

Table S8. Vapor pressure deficit (VPD, kPa) calculated from air temperature and relative humidity inside the five experimental greenhouses for eggplant at four key growth stages (DAT 10, 24, 38, and 52). Values represent the mean ± standard deviation (SD).

DAT	VPD (kPa)				
	G1	G2	G3	G4	G5
10	1.41 ± 0.11	1.24 ± 0.10	1.37 ± 0.11	1.34 ± 0.10	1.29 ± 0.10
24	1.69 ± 0.11	1.68 ± 0.12	1.59 ± 0.12	1.55 ± 0.10	1.50 ± 0.11
38	2.10 ± 0.14	2.01 ± 0.14	1.88 ± 0.13	2.07 ± 0.14	1.92 ± 0.13
52	2.28 ± 0.13	2.32 ± 0.13	2.20 ± 0.12	2.23 ± 0.13	2.11 ± 0.13

Table S9. Average air temperature (T, °C) recorded inside the five experimental greenhouses for lettuce at four key growth stages (DAT 10, 24, 38, and 52). Values represent the mean ± standard deviation (SD).

DAT	T (°C)				
	G1	G2	G3	G4	G5
10	21.9 ± 0.5	21.7 ± 0.5	21.5 ± 0.4	21.8 ± 0.5	22.1 ± 0.5
24	19.1 ± 0.5	18.8 ± 0.5	19.4 ± 0.4	19.3 ± 0.5	19.2 ± 0.5
38	15.2 ± 0.4	14.5 ± 0.4	14.7 ± 0.3	14.5 ± 0.3	14.6 ± 0.4
52	12.8 ± 0.4	12.8 ± 0.3	12.5 ± 0.4	12.1 ± 0.3	12.2 ± 0.3

Table S10. Average relative humidity (RH, %) recorded inside the five experimental greenhouses for lettuce at four key growth stages (DAT 10, 24, 38, and 52). Values represent the mean ± standard deviation (SD).

DAT	RH (%)				
	G1	G2	G3	G4	G5
10	65 ± 3	68 ± 3	66 ± 3	64 ± 3	65 ± 3
24	67 ± 3	70 ± 3	67 ± 3	69 ± 3	69 ± 3
38	73 ± 3	72 ± 3	72 ± 3	71 ± 3	73 ± 3
52	72 ± 3	73 ± 3	70 ± 3	70 ± 3	72 ± 3

Table S11. Vapor pressure deficit (VPD, kPa) calculated from air temperature and relative humidity inside the five experimental greenhouses for lettuce at four key growth stages (DAT 10, 24, 38, and 52). Values represent the mean ± standard deviation (SD).

DAT	VPD (kPa)				
	G1	G2	G3	G4	G5
10	0.92 ± 0.06	0.83 ± 0.06	0.87 ± 0.05	0.94 ± 0.06	0.93 ± 0.06
24	0.73 ± 0.05	0.65 ± 0.05	0.74 ± 0.05	0.69 ± 0.05	0.69 ± 0.05
38	0.47 ± 0.04	0.46 ± 0.03	0.47 ± 0.03	0.48 ± 0.03	0.45 ± 0.03
52	0.41 ± 0.03	0.40 ± 0.03	0.43 ± 0.03	0.42 ± 0.03	0.40 ± 0.03

4. Effect size

Table S12. Relative changes (% effect size) of post-harvest traits in eggplant under different UV-B transmittances compared to the reference greenhouse (3% UV-B). Values represent the percentage change of each variable relative to the 3% UV-B control. These data provide a quantitative overview of the effect of UV-B exposure on nutritional and productivity traits.

%UV-B	Yield	HAA	LAA	Vitamin C	Carotenoids	Chlor a+b
3	0	0	0	0	0	0
24	-10.2	+63.3	+48.0	-9.4	-57.1	-8.3
30	-14.0	+62.0	+124.0	-5.8	-78.6	+8.3
35	-5.3	+40.7	+132.0	+36.6	-64.3	+8.3
39	-0.9	+78.0	+77.3	+45.2	-50.0	+8.3

Table S13. Relative changes (% effect size) of post-harvest traits in lettuce under different UV-B transmittances compared to the reference greenhouse (3% UV-B). Values represent the percentage change of each variable relative to the 3% UV-B control. These effect sizes highlight the influence of UV-B exposure on both productivity and nutraceutical quality.

%UV-B	Yield	HAA	LAA	Vitamin C	Carotenoids	Chlor a+b
3	0	0	0	0	0	0
24	+33.8	+6.2	-17.9	+26.1	+38.9	-38.1
30	+27.5	+5.4	-19.0	+51.5	+100.0	+55.6
35	+35.1	+44.0	+79.5	+14.2	+11.1	+4.8
39	+13.2	+1.2	+20.0	+204.0	+61.1	+3.2

5. PCA analysis

Table S14. Correlation matrix of post-harvest traits for eggplant, used in the exploratory principal component analysis (PCA). Values indicate Pearson correlation coefficients between each pair of traits, highlighting coordinated responses among productivity and nutraceutical parameters under different UV-B greenhouse treatments.

	Yield	HAA	LAA	Vitamin C	Carotenoids	Chlor A+B
**Yield**	1	-0,43835	-0,51117	0,5679	0,76331	0,10139
**HAA**	-0,43835	1	0,52595	0,25497	-0,77263	0,17487
**LAA**	-0,51117	0,52595	1	0,37048	-0,88155	0,67765
**Vitamin C**	0,5679	0,25497	0,37048	1	-0,09445	0,642
**Carotenoids**	0,76331	-0,77263	-0,88155	-0,09445	1	-0,33408
**Chlor A+B**	0,10139	0,17487	0,67765	0,642	-0,33408	1

Table S15. Loadings of post-harvest traits for eggplant PCA. Values represent the contribution of each trait to the first two principal components, providing insight into which variables drive the observed variation among UV-B treatments.

	PC1	PC2
**Yield**	-0,34109	0,54923
**HAA**	0,43154	-0,07472
**LAA**	0,52789	0,09932
**Vitamin C**	0,16705	0,64689
**Carotenoids**	-0,5404	0,17247
**Chlor A+B**	0,31436	0,48445

Table S16. Correlation matrix of post-harvest traits for lettuce, used in the exploratory principal component analysis (PCA). Values indicate Pearson correlation coefficients between traits, showing coordinated relationships between productivity and antioxidant-related traits under varying UV-B transmittances.

	Yield	HAA	LAA	Vitamin C	Carotenoids	Chlor A+B
**Yield**	1	0,6075	0,22759	-0,19753	0,26268	-0,05365
**HAA**	0,6075	1	0,86604	-0,33445	-0,3564	-0,00463
**LAA**	0,22759	0,86604	1	0,00429	-0,47909	-0,0454
**Vitamin C**	-0,19753	-0,33445	0,00429	1	0,47179	0,09749
**Carotenoids**	0,26268	-0,3564	-0,47909	0,47179	1	0,60665
**Chlor A+B**	-0,05365	-0,00463	-0,0454	0,09749	0,60665	1

Table S17. Loadings of post-harvest traits (Yield, HAA, LAA, Vitamin C, Carotenoids, and Chlorophyll a+b) for lettuce PCA. Values indicate the contribution of each trait to the first two principal components, illustrating which variables primarily drive variation among UV-B greenhouse treatments.

	PC1	PC2
**Yield**	0,2798	0,51157
**HAA**	0,5741	0,31627
**LAA**	0,51147	0,1644
**Vitamin C**	-0,30975	0,17519
**Carotenoids**	-0,43177	0,55685
**Chlor A+B**	-0,2194	0,52006
